# Impact of ureteral access sheath on renal stone treatment: prospective comparative non-randomised outcomes over a 7-year period

**DOI:** 10.1007/s00345-019-02878-5

**Published:** 2019-07-24

**Authors:** Ashleigh Lima, Thomas Reeves, Robert Geraghty, Amelia Pietropaolo, Lily Whitehurst, Bhaskar K. Somani

**Affiliations:** Department of Urology, University Hospital Southampton NHS Foundation Trust, Wessex Clinical Research Network and Simulation Lead for Urology, Tremona Road, Southampton, SO16 6YD UK

**Keywords:** Access sheath, Calculi, Laser, Ureteroscopy, RIRS

## Abstract

**Purpose:**

To compare the outcomes (stone free rate and complications) of renal stone treatment with and without the use of ureteral access sheath (UAS). The worldwide use of UAS has risen over the last decade; however, questions still remain on the safety and outcomes with its use. We wanted to look at the role of UAS for treatment of consecutive renal stones over a 7-year period.

**Methods:**

The outcomes of flexible ureteroscopy and stone treatment (FURS) for renal stones with and without the use of UAS was prospectively compared from March 2012 to July 2018. Patients were divided into two groups: group-1 where UAS was used for stone treatment and group-2 where a UAS was not used. Data were collected prospectively on consecutive patients for demographics, stone size, location and number, pre and post-operative stent usage, operative time duration, stone free rate (SFR), length of stay and complications.

**Results:**

During the study period, 338 patients underwent FURS for renal stones, of which a UAS was used for 203 (60%) patients. The mean age of patients was 56 years (range 2–89 years) with a male:female ratio of 204:134. The mean cumulative stone size and the mean number of stones was 16.5 ± 10.8 mm and 11.37 ± 8.08 mm (*P* < 0.001), and 2.17 ± 1.99 and 1.66 ± 1.50 (*P* = 0.009) for groups 1 and 2 respectively. The pre and post-operative stent insertion rates were similar in the two groups. The procedural time was longer in group-1 (54.8 ± 25.8 min) compared to group-2 (41.3 ± 22.2 min) (*P* < 0.001). The SFR for group-1 (88%) was slightly lower than group-2 (94%) although this was not statistically significant (*P* = 0.07). There were no intra-operative complications in either of the groups. Post-operative complications were seen in eight patients in group-1 (7 Clavien I/II and 1 Clavien IVa) and two patients in group-2 (Clavien I) (*P* = 0.19).

**Conclusion:**

The use of UAS for renal stones is safe with no intra-operative complications noted in our series. Good stone-free rates were obtained for large and multiple renal stones with a small risk of minor complications post-operatively.

## Introduction

Ureteric Access Sheath (UAS) are commonly used in endourological procedures especially during flexible ureteroscopy (FURS). They aid in facilitating multiple passage in the kidney, improving irrigation with better fluid outflow thereby improving vision and decreasing the intrarenal pressure [1–9]. There is a perceived advantage of minimising scope damage with it [3]. Conversely, it has been suggested that ureteral wall injuries occur more frequently during ureteroscopy (URS) when using UAS is used [4]. Recorded post-operative complications of FURS with UAS use include ureteric perforation, ischaemia from decreased ureteral blood flow and stricture formation [5].

The use of UAS is widely recognised as a technique utilised to keep the intrarenal pressure low when performing FURS for larger and more complex stones, although some authors advocate its use for most renal stones [6–8]. Managing intrarenal pressure seems to prevent acute kidney injury and possibly also reduces the risk of sepsis [10–14]. Operative times greater than 60 min with irrigation pressure > 20 cmH_2_O is linked with renal damage, which is further worsened by peak pressures during forced irrigation, all of which is partly negated with the use of UAS [12]. There is some debate on the size of UAS sheath used with a trend towards using smaller diameter UAS. Although sheath size does influence the irrigation flow, this is much less pronounced when the working channel is occupied [5, 6, 15].

Ureteric perforation and fluid extravasation can lead to delayed stricture formation especially in the presence of infection [12]. However, a recent study from Anbarasan et al. analysed FURS with UAS in paediatric population and found excellent stone free rate (SFR) with no intra or post-operative complications and no incidence of ureteric stricture on a 26-month follow-up [16]. Although UAS is commonly used in clinical practice, no formal guidelines currently exist [17].

While the size and length of UAS vary depending on the pre-existing ureteral compliance and presence of pre-operative stent, its clinical use usually depends on the surgeon’s discretion and patient’s anatomy [11]. Advances in technology including miniaturisation of endoscopes will reduce the standard UAS diameter, thereby avoiding the use of oversized UAS. This will prevent complications caused by kinking, buckling and forced insertion [18]. Advances and standardisation of a step-wise technique will further reduce intra operative complications [19, 20].

There remains a lack of consensus as to the true impact of UAS on surgical outcomes for renal stones. We hypothesise that the use of an access sheath helps to achieve good SFR for treatment of renal stones without increasing the complication rates. This study aims to report the outcomes of ureteroscopy for treatment of renal stones using an access sheath from a specialist endourology centre in the United Kingdom.

## Methods

Prospective outcomes were recorded for consecutive patients undergoing FURS for renal stones over a period of 7 years between March 2012 and July 2018. The audit was registered in the hospital ‘Clinical effectiveness and audit office’. The inclusion criteria were the presence of renal stones of any size or location but patients with ureteric stones or combined renal and ureteric stones were excluded. Outcomes were collected for patients wherein the surgery was performed or supervised by a single surgeon (BS) and analyzed by a third party (RG) not involved in the original procedure. Patients were divided into two groups based on the use of access sheath. Data analysed were patient demographics, stone size, number of stones, length of inpatient stay, operative time, pre- and post-operative stent placement, stone location, stone free rate and post-operative complications. Complications were classified according to the Clavien–Dindo classification [21]. Data were recorded on an excel spreadsheet (Microsoft, USA) and analysed using SPSS version 24. Results are displayed with *P* values and 95% confidence intervals (CI), using Chi squared and Fisher’s exact test (FET). A *P* value < 0.05 was considered statistically significant.

### Technique

The ureteroscopy procedure was performed according to our previously described and validated technique which is used for both endoscopic simulation teaching and clinical use; rigid cystoscopy and placement of a safety guidewire, semi-rigid ureteroscopy over a working guidewire, placement of an access sheath, and flexible ureterorenoscopy thereafter [16, 19, 20, 22].

Patients were placed in the lithotomy position under general anaesthesia and given appropriate antibiotic cover. The bladder and ureteric orifice were directly visualised by rigid cystoscopy prior to the introduction of a safety guidewire. Following this, a semi-rigid ureteroscopy was performed over a second working wire up to the pelvi-ureteric junction (PUJ) or as far proximally as safely achievable, allowing passive dilatation of the ureteric orifice and ureter. This helped in calibration of the ureter to judge whether an access sheath could be inserted, and an estimate of which size UAS could be safely accommodated. Appropriately sized UAS was then inserted over the working wire and positioned just distal to the PUJ under fluoroscopic guidance. Based on the findings of semi-rigid URS where the ureter was judged to be tight and it was felt that the UAS could not be safely inserted, the FURS was inserted radiologically over a safety wire. The ureter was inspected for damage on withdrawal of the FURS. Stents were placed postoperatively based on the clinical judgement after finishing the procedure.

### Equipment

The access sheath used was a 9.5/11F or 12F/14F or 14F/16F Cook Flexor UAS sheath (Cook Medical, USA) wherein a size 45 cm length was used for males, a 35 cm length was used for females and paediatric patients. Flexible ureteroscopy was done using a 7.5F Flex X2 flexible ureteroscopes [Karl Storz Endoscopy (UK) Ltd.,Slough, UK] with a holmium:YAG laser [20 W or 100 W; Lumenis (UK) Ltd., Elstree, UK] using a 272 micron laser fiber (Lumenis, Inc.). Stone fragment was removed for stone analysis using a Cook NGage nitinol stone extractor (Cook Medical, USA). A 6F ureteric stent (Cook Medical or Coloplast) was inserted post-operatively in majority of patients and removed a few weeks postoperatively.

### Diagnosis and follow-up

The stone diagnosis was established using a non-contrast CT scan (NCCT) with follow-up based on plain x-ray for radiopaque stones and ultrasound (USS) for radiolucent stones 2–3 months post-ureteroscopy. Stone-free rate was defined using a combination of being endoscopically stone free immediately after FURS and radiologically stone free (defined as fragments ≤ 2 mm) on follow-up imaging [22].

Patients were divided into two groups: group-1 where UAS was used for stone treatment and group-2 where a UAS was not used.

## Results

A total of 338 patients underwent FURS for renal stones, of which UAS was used in 203 (60.1%) patients (Table 1). The access sheath used were 9.5F/11.5F (*n* = 89, 44%), 12F/14F (*n* = 110, 54%) and 14F/16F (*n* = 4, 2%). The mean age of patients was 56 years (range 2–89 years) with a male:female ratio of 204:134. The mean cumulative stone size and the mean number of stones was 16.5 ± 10.8 mm and 11.37 ± 8.08 mm (*P* < 0.001), and 2.17 ± 1.99 and 1.66 ± 1.50 (*P* = 0.009) for groups 1 and 2, respectively.

**Table 1:** Patient demographics and outcomes for outcomes of renal stones with and without the use of UAS (ureteral access sheath)

FURS for renal stones (*N* = 338)	Group-1 UAS used (*n* = 203)	Group - 2 UAS not used (*n* = 135)	*P* (95% CI) [Test]
Mean Age	55.0 ± 20.3	58.1 ± 17.8	*P* = 0.15 (− 1.12 to 7.33)
Gender (*n*)	Male: 117	Male: 87	*P* = 0.21
	Female: 86	Female: 48	
Mean cumulative stone size (mm)	16.5 ± 10.8	11.37 ± 8.08	*P* < 0.001 (3.00–7.27)
Mean number of stones	2.17 ± 1.99	1.66 ± 1.50	*P* = 0.009 (0.13–0.89)
Mean length of stay (days)	0.82 ± 5.06	0.22 ± 0.75	*P* = 0.10 (− 1.31 to 0.12)
Mean operative time (min)	54.8 ± 25.8	41.3 ± 22.2	*P* < 0.001 (8.00 to 19.07)
Pre-operative stent, *n* (%)	56 (27.6%)	38 (28.3%)	*P* = 0.88
Post-operative stent, *n* (%)	164 (82.4%)	107 (81.1%)	*P* = 0.77
Stone location, *n* (%)	LP: 81 (39.9%)	LP: 60 (44.4%)	*P* = 0.66
	MP: 24 (11.8%)	MP: 14 (10.4%)	
	UP: 15 (7.4%)	UP: 13 (9.6%)	
	RP: 28 (13.8%)	RP: 20 (14.8%)	
	Renal NS: 55 (27.1%)	Renal NS: 28 (20.7%)	
Stone free, *n* (%)	177 (88.1%)	125 (94.0%)	P = 0.07
Complications, *n* (%)	8 (4%)	2 (1.5%)	P = 0.19
	1 stent pain (Cl I)	1 clot colic (Cl I)	
	6 urosepsis (Cl II)	1 stent pain (Cl I)	
	1 urosepsis (Cl IVa)		

*LP* lower pole, *MP* mid pole, *UP* upper pole, *RP* renal pelvis, *NS* not-specified, *Cl* Clavien

The pre-operative stent insertion rates were 27.6% and 28.3% with the post-operative stent insertion rates of 82.4% and 81.1% in groups 1 and 2, respectively.(Table 1). The procedural time was longer in group-1 (54.8 ± 25.8 min) compared to group-2 (41.3 ± 22.2 min) (*P* < 0.001). The SFR for group-1 (88%) was slightly lower than group-2 (94%) although this was not statistically significant (*P* = 0.07).

There were no intra-operative complications in either of the groups. Eight patients experienced post-operative complications in group-1 with one stent pain (Clavien I), six urinary tract infection or urosepsis (Clavien II) and one urosepsis needing intensive care unit admission (Clavien IVa). Only two patients in group-2 had post-operative complications (Clavien I) with one clot colic and stent related pain each.

## Discussion

To the author’s knowledge, this is the largest single-centred prospective study looking at the outcome of FURS for renal stones with and without the use of UAS. While the use of UAS achieved excellent SFR with a low risk of post-operative complications, no intra-operative complications were noted with its use. This was partly down to the routine use of semi-rigid URS prior to placing an access sheath allowing passive ureteric dilatation and partly because UAS was not used if the ureter was judged to be tight for a safe insertion of UAS. Similarly, a clinical judgement was made on the size of UAS that could be safely placed. When we compared the two groups, UAS was used in patients with larger or multiple renal stones although there was no difference in the rates of post-operative stent insertion rates between the two groups. This perhaps reflects a clinical bias in the use of UAS for larger more complex renal stones whereas for smaller stones it was not routinely used. The overall SFR with the use of UAS was 88% and the post-operative complications were 4%, with only one Clavien IVa complication related to urosepsis.

In a previous study, Geraghty et al. looked at the role of UAS for large renal stones with an overall complication rate of 6%, although they found no difference in the SFR or complication rates with and without the use of UAS [23]. The Clinical Research Office of the Endourological Society (CROES) study has been the largest study to date, involving 1494 patients undergoing FURS with UAS [24]. Although no difference in SFR was noted, there was an overall reduction in infection related complications (fever, UTI, sepsis) with the use of UAS. A previous study from Israel showed a failure rate of insertion in 22% with the use of 14F stent and showed a higher success rate in patients with pre-operative ureteric stent, a prior history of ureteroscopy and in the elderly age group [25]. In another retrospective study by Baş et al. the use of UAS was not associated with complications although stone size, multiplicity and congenital renal abnormality were associated with it [26]. Successful placement of UAS was possible in 91% of patients in a retrospective study of 316 patients by Giusti et al. [27]. After a follow-up of 1-month, the SFR was 79%, the complication rates were 29% of which the majority were Clavien I/II complications.

A previous systemic review and meta-analysis by Huang et al. showed no difference in SFR, operative time and intra-operative complications with the use of UAS although the post-operative complications were slightly higher with its use [28]. Our prospective data of consecutive patients with renal stones show similar results in both groups although with a slightly higher operative time in patients with UAS owing to higher stone size, multiplicity and the time taken to insert the sheath itself.

While intra-operative ureteric injuries are mentioned with the use of UAS there does not seem to be any long-term sequelae [3, 29, 30]. Also, subjective difficulty or longer insertion time for sheath placement were associated with high-grade injury, and in such instances a lower threshold was recommended for using a smaller size sheath [29]. To avoid UAS related injury, we recommend a routine semi-rigid ureteroscopy over an access guidewire in all cases prior to performing a flexible ureteroscopy which could be done with or without an access sheath [19, 20]. The semi-rigid URS allows passive dilatation of the ureter and allows an estimate on whether it is possible to place a UAS [16]. The latter is only placed if the ureter would allow it, otherwise a FURS is guided over a working guidewire.

Our complication rates with the use of UAS was 4% but no intra-operative complications were noted compared to CROES global study where 6% had intra-operative complications including a ureteral perforation rate of 1% [24]. Although our study includes all patients with renal stones, we did not report on how many patients had a trial of UAS where it was unsuccessful, which was around 1% in the CROES study. Similarly, being a non-randomised study, patients where UAS was used had larger and multiple renal stones compared to the group where UAS was not used. The imaging modality used for follow-up was mainly plain X-ray or USS rather than a CT scan. The follow-up period was short and perhaps not enough to identify any potential patients with ureteric stricture. Recent research has found that UAS placed under direct or endoscopic vision reduced operative time and radiation exposure to the patient and surgical team, although this work needs further validation [31, 32]. More work also needs to be done on the use of alpha blockers which seems to increase the success with placement of UAS and this needs to be incorporated in future trials [33].

## Conclusion

The use of ureteral access sheath for renal stones is safe with no intra-operative complications noted in our series. Although good stone free rates were obtained in both groups with and without the use of UAS, patients with UAS use had larger or multiple renal stones.
